# The disproportionate burden of HIV and STIs among male sex workers in Mexico City and the rationale for economic incentives to reduce risks

**DOI:** 10.7448/IAS.17.1.19218

**Published:** 2014-11-14

**Authors:** Omar Galárraga, Sandra G Sosa-Rubí, Andrea González, Florentino Badial-Hernández, Carlos J Conde-Glez, Luis Juárez-Figueroa, Sergio Bautista-Arredondo, Caroline Kuo, Don Operario, Kenneth H Mayer

**Affiliations:** 1 Brown University School of Public Health, Providence, RI, USA; 2 Instituto Nacional de Salud Pública (INSP), Cuernavaca, Mexico; 3 Clínica Especializada Condesa, Mexico City, Mexico; 4 Psychiatry and Mental Health, University of Cape Town, Cape Town, South Africa; 5 The Fenway Institute, Beth Israel Deaconess Medical Center, Boston, MA, USA; 6 Harvard Medical School, Boston, MA, USA

**Keywords:** male sex workers, men who have sex with men, conditional cash transfer, conditional economic incentives, HIV/STI prevention, risk premium, compensating differential, Mexico

## Abstract

**Introduction:**

The objective of this article is to present the rationale and baseline results for a randomized controlled pilot trial using economic incentives to reduce HIV and sexually transmitted infection (STI) risk among male sex workers (MSWs) in Mexico City.

**Methods:**

Participants (*n*=267) were tested and treated for STIs (chlamydia, gonorrhoea, syphilis and HIV) and viral hepatitis (hepatitis B and C), received HIV and STI prevention education and were randomized into four groups: (1) control, (2) medium conditional incentive ($50/six months), (3) high conditional incentive ($75/six months) and (4) unconditional incentive ($50/six months). In the conditional arms, incentives were contingent upon testing free of new curable STIs (chlamydia, gonorrhoea and syphilis) at follow-up assessments.

**Results:**

Participants’ mean age was 25 years; 8% were homeless or lived in a shelter, 16% were unemployed and 21% lived in Mexico City less than 5 years. At baseline, 38% were living with HIV, and 32% tested positive for viral hepatitis or at least one STI (other than HIV). Participants had a mean of five male clients in the previous week; 18% reported condomless sex with their last client. For 37%, sex work was their main occupation and was conducted mainly on the streets (51%) or in bars/discotheques (24%) and hotels (24%). The average price for a sex transaction was $25 with a 35% higher payment for condomless sex.

**Conclusions:**

The findings suggest that economic incentives are a relevant approach for HIV prevention among MSWs, given the market-based inducements for unprotected sex. This type of targeted intervention seems to be justified and should continue to be explored in the context of combination prevention efforts.

## Introduction

Mexico, a country of 112 million people, has a concentrated HIV epidemic [1] with an HIV prevalence of 0.3% in the adult population (15–49 years), 16.9% among men who have sex with men (MSM), and 18.2% among male sex workers (MSWs) [2,3]. The elevated HIV risk among MSM has been associated with behavioural factors including multiple sexual partners and inconsistent condom use [4–12]. Other factors, such as poverty and economic insecurity, also determine behavioural risks; and, as such, interventions that alleviate the socioeconomic pressures that underpin behavioural risk taking can reduce HIV and other sexually transmitted infections (STIs) among MSWs [13–19] .

Conditional cash transfer (CCT) interventions are a type of economic intervention that incents specific behaviours linked to positive outcomes such as improved health [20]. CCT interventions have been implemented in generalized HIV epidemic settings (Tanzania and Malawi) and have proved effective in reducing HIV and STI risks and prevalence [21,22]. Similarly, CCTs have been used in Mexico since the late 1990s through programmes that provide incentives to poor families to keep school-aged children in school and attend preventive health check-up visits [23]. No efforts in Mexico, however, have directly linked incentives for HIV and STI prevention, including regular STI medical check-ups and treatment, targeting populations at high risk. This novel approach shows promise in light of Mexico's history of economic interventions to promote public health, and its concentrated HIV epidemic disproportionately affecting MSM. This article describes an effort to adapt a CCT intervention to the context of HIV and STI prevention services for economically disadvantaged MSWs in Mexico City.

Interventions to incent safer sexual behaviours can counteract the market-based inducements that sex workers receive from clients to engage in increased risk (i.e., condomless sex). This risk premium (as it is called in economics) has been documented among female sex workers in India, Mexico and Bangladesh [24–26] and among MSWs in Ecuador [27]. Avoiding clients who pay higher prices for unprotected sex implies monetary losses for MSWs. Thus, compensation in the form of economic incentives for self-protection can offer one method for reducing risk behaviours and improving individual and population health.

This article describes the developmental process and rationale for a CCT intervention for HIV and STI prevention. The Punto Seguro (“Secure Point”) intervention involves a randomized controlled design to pilot test whether CCT can reduce HIV risks among MSWs. To our knowledge, this is the first systematic adaptation of CCT of this nature. The rationale for working with MSWs is that they are a vulnerable population with high rates of STI and HIV infection or at high risk of contracting these diseases. Furthermore, male-male transactional sex has been identified as an important driver of the concentrated epidemic in Mexico. Currently, no effective HIV prevention or social programmes exist for this key population in Mexico City. Another innovative aspect is that the levels of the incentives tested were estimated via willingness-to-accept methods [28]. Thus, the aims of this article are to (1) describe the sociodemographic characteristics of the Punto Seguro cohort, (2) present the baseline HIV and STI risk and prevalence rates and (3) analyse commercial sex transaction prices, exploring if a risk premium exists for MSWs in Mexico City which might further drive unprotected sex with paying male clients.

## Methods

### Setting

This research was conducted in Mexico City at Clínica Condesa, which started providing HIV specialized care in 2000. The primary purpose of Clínica Condesa is to provide medical assistance to the Mexico City population who is living with HIV and of low socioeconomic status; prevention is also an important part of its mission. An initiative to expand HIV testing and counselling services for key populations at high risk of infection has been implemented since 2009. Free treatment for HIV and other STIs is included as a service for the target population.

### Participants: inclusion and exclusion criteria

The inclusion criteria were as follows: men; 18–40 years of age; and self-identified as MSWs, or as MSM who had penetrative or receptive anal sex with another man in exchange for money in the last six months and who had 10 or more male sexual partners in the last month. The exclusion criteria were unwillingness to sign an informed consent form to participate in the study, inability to read or speak Spanish and/or inability to respond to the screening questionnaire because of the influence of drugs or alcohol. Transgender individuals were excluded from this study because Clínica Condesa has a separate programme for them. Participants were recruited by trained research staff through direct outreach to community sites where MSW congregate, identified from previous studies [17,28]. Participants also were recruited from within the Clínica Condesa HIV Testing Clinic, for example after using voluntary counselling and testing services and being referred to the research team. We had strict procedures with separated roles (for clinical and research categories) ensuring an understanding that regular clinic services would not be denied regardless of a participant's decision to enrol in the study.

We used several strategies for ensuring recruitment from a diverse sample: the recruitment team (a) regularly visited the sites where sex workers congregate, mainly La Alameda, and a visit was made at least once a week during the busiest times so that participants could be invited; (b) provided transportation to participants so that they could more easily obtain check-ups and STI test results from the clinic; (c) involved qualified personnel (a health counsellor and psychologist) with several years of experience working with the community; and (d) set up a referral process to recruit eligible individuals who were already attending Clínica Condesa.

### Data collection

Data collection took place in collaboration with the Mexican National Institute of Public Health (INSP) and Consortium for HIV/AIDS and TB Research (CISIDAT). All participants were interviewed at Clínica Condesa. Interviews took place in a private area using portable laptop computers with audio computer-assisted interviewing (A-CASI) questionnaires. Electronic records were de-identified, and only code numbers were used for data analysis. Identifiable private information was available only to clinical staff (and used solely for follow-up and treatment referral purposes).

The *primary outcomes* were self-reported condom use (with last three clients; as well as intentions for condom use [29]) and self-reported number of sexual partners (commercial and non-commercial). The *secondary outcomes* were self-reported STIs and commercial sex transaction prices (for protected and unprotected sex episodes, by type of sexual act). *Biological outcomes* were as follows: testing in all groups was conducted for chlamydia, gonorrhoea, syphilis, hepatitis B, hepatitis C and HIV.

Participants provided blood and urine samples for testing. Specimens were collected under standard bio-safety protocols by trained staff and analysed by lab technicians. Urine specimens were collected and tested for gonorrhoea and chlamydia (PCR Cobas-Amplicor; Roche, Basel, Switzerland), and blood specimens served to measure the presence of HIV, hepatitis B, hepatitis C and syphilis antibodies (Abbott HIV-1 and HIV-2, Ag/Ab Combo, anti-HBc, anti-HCV and syphilis TP quimioluminiscence immunoassay (Abbott Laboratories, North Chicago, IL, USA) running in Architect i2000 (Abbott); HIV-positive samples were confirmed with HIV-1 and HIV-2 CombFirm (Orgenics, Yavne, Israel); and anti-HBc+ was tested with Determine HBsAg and syphilis TP+ (Abbott) with tittered VDRL (the Venereal Disease Research Laboratory test)). At the baseline survey, two subgroups were defined for the markers of syphilis and hepatitis B: antibody positivity was regarded as a lifetime marker of past or present infection, whereas treponemic antibody positivity together with VDRL demonstrated active syphilis, and anti-HBc plus HBsAg positivity indicated current hepatitis B virus infection. All participants received results from trained male health care providers certified in STI and HIV counselling, and any participant with positive results for any STI was referred for treatment (free of charge) at Clínica Condesa following the standard guidelines for counselling, testing and treatment of HIV and STIs in Mexico. Participants living with HIV and/or viral hepatitis were still eligible to stay free of new curable STIs (chlamydia, gonorrhoea and syphilis), and they could still receive the incentives if they had been randomized to an incentive arm. All participants provided informed consent, and all procedures were approved by Institutional Review Board (IRB) committees at the Mexican National Institute of Public Health (INSP) and at Brown University.

### CCT adaptation

This intervention is part of a randomized controlled pilot, Punto Seguro. The pilot trial has four arms: control group, medium conditional incentives (600 pesos, or about $50, every six months), high conditional incentives (900 pesos, or about $75, every six months), and unconditional incentives (600 pesos every six months). Participants in the conditional arms receive the incentive amount if they tested negative for new curable STIs (chlamydia, gonorrhoea and syphilis) at months 6 and 12. In the unconditional arm, they receive the medium incentive regardless of new curable STI status. The amounts of the incentives were established using an economic approach for willingness-to-accept (WTA) [28]. We used a computer experiment to measure the optimal level of incentives by interviewing 1,745 MSM and MSWs, and we found that at a rate of $288 a year, more than three-quarters of the men would attend prevention talks, engage in testing for STIs and attempt to stay free of STIs. To obtain a similar level of participation among the subsample who were MSWs, the price was lower: $156 a year. Thus, potential participants felt that these amounts were relevant, based on previous research. Also, to limit any potentially negative framing effects, we avoided the term “low incentive” and always referred to medium and high incentives. Moreover, we also purposely tried to keep the incentive levels as low as possible with the issues of sustainability and potential cost-effectiveness in mind. Given the high costs of HIV treatment in Mexico [30] of about $5,000 to $7,000 per person per year, if the intervention helps individuals avoid HIV infections, then there may be cost savings in the long run. Because cash may have unintended consequences, such as increased use of alcohol and drugs, we used vouchers for food and groceries as a form of payment when the conditionality was fulfilled, as well as for the inconvenience fees and the unconditional incentive payments. Hence, we called the intervention “conditional economic incentives” (CEIs) instead of CCT.

### Statistical procedures and analysis

We first present descriptive statistics for sociodemographic data (Table 1) as well as STI and HIV risks (Table 2), showing the sample sizes responding to each specific question. Next, we present cross-tabulation tables of self-report versus actual STI/HIV status (Table 3). We also report commercial sex transactions during the past week in terms of the highest price, the lowest price and the average price, as well as for the last three clients (Table 4). Given the participants’ estimations of highest, lowest and average prices in the past week, we report the mean, standard deviation, median, minimum and maximum. We placed more emphasis on the median results because of the presence of large outliers.

**Table 1:** Baseline sociodemographic characteristics

Sociodemographic variables	*N*	*n*	% Mean (SD)
Age (years)	253		**24.7 (4.68)**
Schooling	250		
1–6 years		31	12.4
7–9 years		73	37.6
10–12 years		94	20.8
13 or more years		52	37.3
Marital status	252		
Married/free union		50	19.8
Single		199	78.9
Separated/widower		3	1.19
Main occupation now	242		
Work (non-sexual)		54	22.3
Unemployed		39	16.1
Student		36	14.8
Sex work		90	37.1
Own business		12	4.96
Other		11	4.55
Housing	245		
Own an apartment or home		65	26.3
Rent an apartment or home		99	40.4
Staying at friend's house or apartment		25	10.2
Rent room (hotel, motel, pension)		35	14.2
On the street		13	5.31
Other		8	3.26
Has been in Mexico City less than 5 years	211	45	21.3
Does not have health insurance	238	124	52.1
Sex work location	186		
Bar/disco/pub		45	24.1
Hotel/motel		45	24.1
Street/public park		94	50.5
Sex club		6	3.23
Beauty salon		5	2.69
Massage parlour		9	4.84
Sauna/bath house		13	6.99
Other		11	5.80

Table presents percentages, unless otherwise noted.

*N*=total respondents; *n*=responses for specific question; SD=standard deviation.

**Table 2:** Baseline HIV and STI risk and prevalence

HIV and STI risk and prevalence	*N*	*n*	% Mean	SD
Number of sexual partners last week				
Non-paying male	116		2.76	4.03
Non-paying female	39		2.02	4.00
Male clients	102		4.55	4.98
Female clients	15		2.53	6.03
Condom was used in …				
Last commercial encounter	216	177	81.9	–
Second-to-last commercial encounter	202	168	83.7	–
Third-to-last commercial encounter	195	153	78.4	–
Has ever had HIV test	261	203	77.7	–
Knows that healthy-looking person can transmit HIV	259	227	87.6	–
Oral or anal sex with another man before age 15	232	81	34.9	–
First compensated sex before age 15	188	36	19.4	–
Body mass index (kg/m^2^)	230		23.8	5.43
Sexual services with last client	139			
Penetrative sex		46	33	
Receptive sex		37	26.6	
Penetrative and receptive sex		23	16.5	
Masturbation		31	22.3	
Oral sex		74	53.2	
Biological test results	267			
Active syphilis		55	20.7	–
Chlamydia		26	9.81	–
Gonorrhoea		6	2.26	–
Active hepatitis B		8	3.01	–
Hepatitis C		3	1.13	–
Has HIV (with confirmatory test)		100	37.7	–
Has any STI		85	31.8	–

Table presents percentages, unless otherwise noted.

SD=standard deviation; STI=sexually transmitted infection and viral hepatitis (active syphilis, chlamydia, gonorrhoea, and active hepatitis B and C); *N*=total respondents; *n*=responses for specific question.

**Table 3:** Knowledge of STI and HIV infection versus actual biological test results

	HIV status (test results)			
	
	HIV-negative		HIV-positive	
	
HIV status (self-report)	*n*	%	*n*	%
HIV-negative	69	41.3	11	11.0
HIV-positive	0	0.00	59	59.0
Don't know	36	21.5	14	14.0
Don't wish to respond	62	37.1	16	16.0
Total	167	100	100	100
	**STI status (test results)**			
	
	**STI-negative**		**STI-positive**	
	
**STI status (self-report)**	***n***	**%**	***n***	**%**

STI negative	41	22.6	22	25.5
STI positive	10	5.55	7	8.13
Don't know	34	18.8	14	16.3
Don't wish to respond	96	53.0	43	50.0
Total	181	100	86	100

Column percentages may not add up to exactly 100 due to rounding.

STI=sexually transmitted infection and viral hepatitis (active syphilis, chlamydia, gonorrhoea, and active hepatitis B and C).

**Table 4:** Prices charged for commercial sex in the past week and last three clients

Prices charged for commercial sex	*n*	Mean	SD	Min	Max	Median	Median (USD)
What was the highest price charged last week?	121	857	1,045	50	8,000	500	42
What was the minimum price charged last week?	121	320	270	50	1,500	200	17
On average, how much did you charge last week?	97	729	1,043	99	6,000	300	25
How much did you charge to your last client?	114	603	756	50	5,500	400	33
How much did you charge to your second-to-last client?	108	515	458	50	3,500	400	33
How much did you charge to your third-to-last client?	96	551	559	50	3,500	375	31

Amounts given in current Mexican pesos (MNX), unless otherwise noted. Last column converts pesos into USD using an exchange rate of 12 pesos/$1 USD.

SD=standard deviation; STI=sexually transmitted infection and viral hepatitis (active syphilis, chlamydia, gonorrhoea, and active hepatitis B and C); USD=US dollars.

Finally, we analysed the differences between prices for commercial sex with and without a condom (Table 5). In economics, this difference is called a compensating differential or risk premium because sex workers are paid higher prices when they have more risk [27,31], a phenomenon observed in other occupations as well [32]. We follow methods used in the *female* commercial sex markets in Mexico, Ecuador and India [24,25,31] and among MSWs in Ecuador [27]. We estimated a log-linear equation using the log of the commercial sex transaction price as the dependent variable, and a dummy variable (=1 if no condoms were used) as the main explanatory variable. The log transformation helped to linearize the distribution and enabled us to apply linear regression methods.

**Table 5:** Baseline regression analysis for the risk premium

Dependent variable: log (sex work transaction fee, last client)	(1)		(2)		(3)	

	Coef.	SE	Coef.	SE	Coef.	SE
Sex work variables						
No condom used with the last client	0.332*	0.178	0.385**	0.181	0.345*	0.198
Met client at a hotel			0.337*	0.174	0.215	0.294
Met client on the street or park			0.087	0.153	0.106	0.214
Sociodemographic characteristics						
Age					−0.141	0.202
Age squared					0.003	0.003
Schooling (ref: 1–6 years)						
7–9 years					0.448	0.303
10–12 years					0.562*	0.312
13 or more years					0.756**	0.336
Marital status (ref: single)						
Married/free union					0.697	0.24
Without health insurance					0.237	0.175
Homeless					−0.109	0.485
Body mass index (kg/m^2^)					−0.029	0.222
Constant	6.003***	0.075	5.841***	0.143	7.572***	2.413
Observations	101		96		76	
*R* ^2^	0.033		0.07		0.204	

*** p<0.01

** p<0.05

* p<0.1

Coef.=coefficient; SE=standard error.

## Results

Table 1 shows the baseline sociodemographic characteristics of the participants. The mean age was 25 years; 12% of respondents completed one to six years of schooling. Most (79%) were single, but almost 20% were cohabitating with a partner. Sex work was the main occupation for 37% of respondents, but 22% had other work (non-sexual), 16% reported to be unemployed and another 15% reported to be students. Although about a quarter (26%) lived in their own apartment or home, most rented an apartment or home (41%); others rented a room (14%), stayed at a friend's (10%), were homeless (5%) or lived in a shelter (3%). In addition, 21% have lived in Mexico City for less than five years. Most MSWs were primarily street based (working mainly in the Alameda Central and Zona Rosa neighbourhoods in Mexico City) (51%), but a smaller number were hotel based and/or recruited partners via the internet (24%) or were based at bars and discotheques (24%).

Table 2 presents the baseline results for HIV and STI risk and prevalence. The mean number of non-paying male sex partners in the previous week was 2.8; and the mean number of male clients in the previous week was 4.55. Condoms were used for 82% of their last commercial encounters, 84% of their second-to-last commercial encounters, and 78% of their third-to-last commercial encounters. In 33% of encounters, MSWs were the insertive partner; whereas in 27% of the acts, they were receptive. They were both receptive and insertive in 17% of the acts. HIV was the most common prevalent infection (38%), followed by syphilis (21%), chlamydia (10%) and active hepatitis B (3%). The less prevalent STIs were gonorrhoea (2%) and hepatitis C (1%).

Table 3 shows comparisons between self-reported STI and HIV status versus actual status verified by biological testing. Of the 100 participants who were confirmed to be HIV-positive: 14% said they didn't know if they had HIV, 11% said they did not have it and an additional 16% said they would rather not respond. Similarly, of the 86 participants who had any other confirmed STI (other than HIV): 7 (8.13%) said they did not have any STI, 14 (16.3%) said they didn't know if they had an STI and an additional 43 (50%) did not wish to respond.

Table 4 shows that the median for the highest price charged for a sexual transaction in the past week was $42. The median for the lowest price charged last week was $17, and the median for the average price charged last week was $25. The median prices charged to the last three clients ranged from $31 to $33.

Table 5 presents the regressions of the prices paid by the last client (log transformed) on the primary explanatory variable, condomless sex, gradually augmenting the model specification based on the literature. In the first column, we present the unadjusted coefficient on no-condom-used, which was 0.332 (*p*<0.1). Then, in columns 2–3, we expand the model and show the adjusted coefficients controlling for up to 11 covariables. In column 3, the adjusted main coefficient was 0.345 (*p*<0.10). Thus, MSWs received a risk premium of 34.5%; this means that, on average, they got paid 34.5% higher prices for condomless sex.

## Discussion

The results show the rationale for an economic-based HIV and STI prevention intervention among groups at high risk of HIV and STI infection in Mexico City. The MSW sample is generally of low socioeconomic status, with high economic need, and it shows high levels of risk for HIV and STI transmission and acquisition, as evidenced by high prevalence for HIV (38%) and high prevalence for other STIs (32%), important levels of condomless sex (17%) and multiple sex partners (about five male clients and three non-paying partners per week). Moreover, we found evidence of a risk premium in the commercial sex transaction prices: MSWs received more money (35% higher prices) to engage in condomless sex. Thus, the economic rationale to provide financial incentives to increase protection seems well justified.

Of the 100 participants who were living with HIV at baseline, 41% did not know that they were HIV infected or declined to self-report their status (Table 3). Given the high number of sexual partners (over 250 per year) and the rates of condomless sex, the implied potential transmission rates constitute a public health concern [33]. Economic incentives may be an avenue to attract MSWs to screening and medical care in an HIV and STI clinic with a team specifically dedicated to this key population. An intervention with incentives is an important avenue to facilitate access to screening and treatment services in a population who otherwise might not accept or access these services. Similarly, of the participants who knew they were living with HIV, only 40% were on treatment; and, of those, only 61% achieved viral suppression. Thus, interventions to improve access and adherence to antiretroviral therapy (ART) among MSWs living with HIV are also warranted, including, possibly, incentives for linkage to care and viral suppression. This seems particularly feasible in a country such as Mexico, which has universal access to ART; participants at Clínica Condesa have access to ART (free to them) provided through Seguro Popular (the social health insurance programme for those without employer-based insurance coverage).

The extant results show that enrolment of a cohort of MSWs is feasible in the context of HIV and STI prevention in Mexico City, and that Punto Seguro is a culturally appropriate intervention in the local context. Furthermore, in terms of HIV prevalence and risk, our cohort seems to be a population at even higher risk than the average MSW population found in a recent population survey [2,3], although this may be explained in part because of the active recruitment within a clinical site (Clínica Condesa).

Because economic need has been identified as an important reason for engaging in sex work and an important barrier to using condoms consistently, our pilot project will test the potential efficacy of a public health and behavioural economics intervention to reduce HIV and STI risk and vulnerability among MSWs in Mexico City. The baseline results presented here confirm previous formative work in which we asked MSWs to endorse the incentive levels necessary for behaviour change ($156/year) [28]; this would represent, on average, trying to avoid unprotected sex with about six clients over a year. Nevertheless, our method to estimate the acceptable incentive may have captured what participants wanted to receive to commit to attending regular visits for testing, and it may not fully reflect a compensation to avoid the risk premium. As such, some corrections would be needed in the willingness-to-accept estimates before implementing a trial.

Furthermore, the benefits may last longer than the intervention itself because of three main mechanisms at work. First, an MSW who is *negative* for HIV and STIs, and is using condoms consistently, will likely remain free of infection. Second, an MSW who is *positive* for HIV and STIs, and is using condoms consistently, is much less likely to transmit HIV and STIs to sexual partners. Third, an MSW who is positive for HIV, has linked to medical care and ART and has achieved virologic suppression is also much less likely to transmit HIV to partners [34]. This would result in fewer new cases of HIV. Intervention with economic incentives may thus work well as a bridge to help MSWs to link to HIV care and ART. Moreover, it is likely that if the financial incentives are stopped, the participants will have already developed other links to the clinic and start receiving other types of positive outcomes (health, psychological, social etc.) from the care team, so that MSWs may continue their ART and/or follow-up with prevention reduction (both HIV-positive and -negative), regardless of the continuity of economic incentives. We acknowledge, nevertheless, that staying free of new curable STIs may contribute to staying HIV-negative, and it can be another incentive among those who are HIV-negative but not those who are already HIV-positive. The differences across HIV serostatus need to be taken into account in this type of incentive programme. In theory, the motivation to protect sexual partners from infection may differ along a continuum of altruistic intentions. Stratified analyses are needed in future trials to empirically test for differential effects by HIV serostatus.

The study has limitations. First, the relatively limited sample size leads to results for the risk premium which are only borderline significant. Second, the incentives for behaviour change may be small given that the risk premium for MSWs, of about 35%, is higher than what we originally hypothesized based on the previous literature, at levels closer to those observed among female sex workers: 9–23% [24,25,35]. Third, the relative ease with which A-CASI allows participants to refuse to answer, or simply skip ahead to the next question, led to several key variables with missing values. Despite these limitations, the current study shows that we have been able to enrol a substantial number of participants from a key population for which only limited research exists, usually with smaller samples and shorter follow-up periods [36].

## Conclusions

This article provides a strong rationale for the adaptation of an economic incentives intervention for male sex workers in the context of HIV and STI prevention. This first evidence of higher payments for unprotected sex in the male commercial sex market in Mexico is also consistent with the justification for using cash transfers to incent HIV risk reductions through STI screening visits as a means to counteract the market-based inducements to engage in behaviours that are more likely to transmit HIV in this heavily affected population.

The Punto Seguro pilot trial has recruited MSWs, and it is an example of an economic-based HIV prevention intervention targeted to the populations who are most at risk in concentrated epidemic settings. As demonstrated by these initial results, this type of intervention seems to be well justified, and it should continue to be explored in the context of combination prevention efforts for key affected populations.
